# Factors associated with utilization of maternal serum screening for Down syndrome in mainland China: a cross-sectional study

**DOI:** 10.1186/s12913-016-1260-6

**Published:** 2016-01-14

**Authors:** Chuanlin Li, Leiyu Shi, Jiayan Huang, Xu Qian, Yingyao Chen

**Affiliations:** 1 School of Public Health, Key Lab of Health Technology Assessment (Ministry of Health), Fudan University, No.138 Yixueyuan Rd, Post box No. 197, Shanghai, 200032 PRC; 2 Primary Care Policy Center, Johns Hopkins, Baltimore, MD 21205 USA; 3 Collaborative Innovation Center of Social Risks Governance in Health, Shanghai, 200032 PRC

**Keywords:** Health service research, Healthcare utilization, Maternal serum screening, Antenatal screening, Down syndrome

## Abstract

**Background:**

Knowledge of the factors that influence maternal serum screening (MSS) service utilization can be used to develop health policies to promote equitable access to MSS and further diagnostic tests. The purpose of this study was to find the factors associated with utilization of MSS as well as the current status of service utilization in mainland China.

**Methods:**

This was a hospital-based cross-sectional study with respondents interviewed with a questionnaire designed based on Andersen’s behavioral model. Descriptive statistics, univariate analysis, and multilevel logistic regression analysis were used to identify the factors associated with MSS utilization, and to explore potential methods to improve screening uptake.

**Results:**

A total of 8110 women who had given birth within the previous 7 days in one of 111 participating institutions from six provinces in mainland China were interviewed. Approximately 36 % of the participants had used MSS. Women between 20 and 35 years, who resided in urban areas, were educated, were in a stable occupation, who had health knowledge, who attended maternal preparation classes, who had received eight or more prenatal checkups, who were from a region of higher social economic status, and who delivered in a tertiary healthcare institution were significantly more likely to use MSS than their counterparts. As compared with other factors, insufficient education is the single most important demographic factor for service underutilization.

**Conclusions:**

Efforts should not only be made to target the population that underuses MSS, but the overall organization of MSS service delivery should be assessed during policy development to make access to MSS equitable to the entire population of mainland China.

## Background

Down syndrome (DS) is a common chromosomal abnormality that occurs in 14.7 per 10,000 live births in China [1], leading to the birth of approximately 23,000–25,000 Chinese children with DS each year [1]. The lifetime economic burden from a family perspective of each person born with DS in China was estimated, in 2003, to be US$47,000 [2]. Maternal serum screening (MSS), the use of various biochemical markers together with ultrasound in the first or second trimester of pregnancy, was first introduced in mainland China in the 1990s [3]. The aim of MSS is to detect the risk of DS, open neural tube defects and other abnormalities noninvasively. According to the authors’ assessment of the published Chinese medical literature, more than 40 healthcare institutions (from more than half of the 31 provinces in mainland China) provided MSS risk evaluation for DS, trisomy 18, trisomy 13 and related birth defects (BD) in 2003. The common screening program till 2003 was either the double test (alpha-fetoprotein (AFP) and human chorionic gonadotropin (HCG), or free-β-HCG)) or the triple test (AFP+ HCG (or free-β-HCG) + unconjugated estriol), which were performed in the second trimester. The reported false positive rate ranged from 3 % to 13 % in different healthcare institutions before 2003. High-risk pregnant women found during screening would be offered invasive diagnostic tests.

In 2003, the Chinese Ministry of Health (MOH) issued a national regulation for the administration of techniques for prenatal diagnosis, with the aim of enabling prospective parents to identify and manage their BD risk, make informed reproductive choices and increase the safety of delivery. According to the regulation: (1) healthcare institutions providing MSS and invasive diagnostic tests need to meet certain requirements to gain certification; (2) high-risk pregnant women found during screening must be provided with options for invasive diagnostic tests; and (3) both MSS and further diagnostic tests are voluntary and based on pregnant women providing written informed consent.

Dramatic advances have been made in DS screening since 2003, as different screening modalities have been introduced and their sensitivity and specificity improved. MSS has been adopted nationwide for the detection and risk assessment of DS and other BD in China [4].

In 2009, China launched a new round of health system reforms that explicitly stated the Government's role in ensuring equity in the provision of public goods and services. Knowledge of the factors that influence the uptake of MSS can be used to develop effective strategies and health policies to promote equal and easy access to MSS and further diagnostic tests.

Based on Andersen’s behavioral model of health services utilization [5], health behaviors are determined by contextual and individual characteristics. Factors that influence health behaviors can be divided into three categories: predisposing factors, enabling factors, and need factors. Contextual characteristics provide a fundamental effect, and they affect people’s health behavior through individual characteristics. The predisposing factors include demographics, social factors, and health beliefs. The enabling factors include the health system, and organizational and financial factors, while the need factors consist of the perceived and evaluated need. A number of international publications have addressed the factors associated with MSS and further diagnostic test utilization. Among these factors, maternal characteristics, including age, education, income, migrant situation, ethnic background, and health beliefs, were frequently examined. Those residing in rural, poor and western areas of China were identified as vulnerable populations, in need of maternal and infant health services [6]. In additional studies from around the world, regional characteristics, the healthcare provider, the local health system, community support, and the capacity and framework of service delivery were scrutinized [7–14]. Studies have compared MSS utilization between different countries, different healthcare institutions and different time periods, which indicated that the macro-contextual characteristics, including socioeconomic status and health policy arrangements, were also affecting MSS uptake and utilization [15–19]. Factors found from the available publications were consistent with Andersen’s behavioral model.

Many large sample studies on prenatal screening utilization were performed in developed countries, implying there is a need for data on MSS uptake from developing countries. Previous Chinese studies have focused on maternal and infant health service use or MSS and further utilization of diagnostic tests, usually performed in one service facility or in one city with a small sample size. However, China encompasses a vast territory, with a huge population that has regional characteristics with distinct rural and urban communities. This provides contextual factors to study for health service utilization [20]. China’s approaches and strategies to improve MSS utilization under its socioeconomic situation can be a reference for other developing countries.

Based on foreign studies and local experience, the factors influencing MSS utilization in mainland China were hypothesized to be maternal, provider and contextual characteristics. The current study aimed to discover the major factors that influence MSS utilization in mainland China, thereby revealing potential strategies for increasing MSS uptake.

## Methods

### Design, setting and participants

This cross-sectional survey was conducted in mainland China. Two-stage cluster sampling was performed. The first stage consisted of convenience sampling in chosen target cities. Six provinces were carefully selected with sufficient populations so that low, medium, and high socioeconomic levels were represented based on 2006 gross domestic product (GDP) per capita [21]. In each selected province, two cities representing relatively low and relatively high socioeconomic levels were selected based on their per capita GDP. Both urban and rural areas of the sampled cities were covered in the survey.

For the second stage, hospital-based cluster samples of service users were carried out in sampled cities selected above. Stratum random sampling was used, first stratified by the level (primary, secondary, and tertiary) of healthcare institution and then sampled purely randomly within each level. About 15 % of the healthcare institutions providing maternity services (including township health centers, community health centers, family planning centers, maternal and child health (MCH) centers and hospitals) in the sampled cities were chosen. Potential participants in this study were women who had given birth in these institutions in the 7 days prior to the survey. Written informed consent was obtained from each participant prior to the survey.

This study planned to survey approximately 8000 women distributed equally from among the 12 sampled cities. Within a certain city, the number of women to be surveyed was allocated among the participating healthcare institutions based on their delivery numbers in the previous calendar year. The representativeness of the sample, based on the respondents’ ethnicity, family income (divided into urban and rural areas) and education level was checked relative to data from the Census and the Chinese Annual Report.

### Questionnaire

Quantitative data were collected during interviews with the participants using a structured questionnaire in Chinese. The questionnaire design, based on Andersen’s behavioral model, included 16 potential factors that might facilitate or impede MSS utilization. These 16 factors were divided into four broad categories: (1) predisposing factors, including the user's demographics (age, place of residence and ethnicity), social structure (occupation, education and migrant situation) and health belief (attendance at maternal preparation classes); (2) enabling factors, including the user's family resources (health insurance coverage and income) and community support (health information and healthcare institution accessibility); (3) need factors, including the perceived need (self-perception of general health status during current pregnancy) and evaluated need (age risk of BD); and (4) health behavior, including the participant's personal health practice (frequency of routine prenatal checkups), process of medical care (knowledge of DS) and MSS utilization.

Data on two kinds of contextual factors were also collected: 1) local/regional economics and societal situation (levels of GDP per capita of the target province/city, population density of the target city) and 2) local health resource distribution (number of health technicians per 1000 population of the target province).

### Data collection and analysis

The survey was conducted from November 2007 to September 2009 and was administered by local maternal and child health technicians (female) in the participating institutions. Prior to the start of the survey at each institution, the local team received intensive training on interview technique and data collection from the research team. Participants were interviewed face-to-face, and the interview usually took about 10–20 min to complete. The surveying period lasted approximately 2 months in each city.

Data were checked for completeness and inconsistencies, edited, coded, and entered into Epidata 3.0. Descriptive analysis was used for the demographic characteristics of the participants. Univariate analysis was used to find factors associated with MSS utilization. Subsequently, given the hierarchical structure of the dataset (i.e. individuals nested within healthcare institutions, healthcare institutions nested within cities), multivariate analysis was conducted using a hierarchical logistical regression model. Two-sided *p*-values < 0.05 were considered to indicate statistical significance. All statistical analysis was done using SPSS software, version 19.0.

### Ethical approval

Ethical approval for the study was obtained from the Ethics Review Committee of Fudan University School of Public Health. All participants were provided with adequate information about the study so that they could make informed and voluntary decisions for participation. In addition, the participants were assured of the confidentiality of the information they would provide. The local interviewers at each institution were responsible for explaining the study’s purpose, procedures, and potential risks and benefits to the respondents.

## Results

### Demographic characteristics of the study population

The questionnaire interview was conducted in 111 healthcare institutions located in 12 cities chosen from six provinces in mainland China. There were 55 general hospitals, 47 MCH centers or specific hospitals, and nine township health centers, community health centers and family planning centers. There were 23 tertiary and 57 secondary healthcare institutions, and 60.4 % (67) of the healthcare institutions were located in urban areas. A total of 8110 women from these institutions participated in this study.

As summarized in Table 1, the mean (± SD) age of the respondents was 26.97 ± 4.64 years, and the majority were younger than 35 years (7581, 93.5 %). The proportion of ethnic minorities was 8.6 % (700), and 62.7 % (5086) of the respondents were employed before delivery. More than half of the respondents had not completed high school; 73.7 % (5981) of the respondents were covered by health insurance schemes, and 62.9 % (5099) of the respondents resided in the countryside. Among respondents, 3752 (46.3 %) reported a per capita household monthly income less than 1200 Yuan RMB, whereas 1670 (20.6 %) reported a per capita household monthly income more than 2400 Yuan RMB (about 320 USD in 2007).

**Table 1:** Unweighted summary statistics of demographic and MSS utilization variables among respondents (*n* = 8110)

Variable		Number	MSS utilization	
			Number(%)	*P* value
Contextual factors (Province / city level)				
	Social economic situation of provinces			
	High	2725	1516 (55.6)	0.000
	Middle	2698	827 (30.7)	
	Low	2687	561 (20.9)	
	Social economic situation within provinces			
	High (higher than the province’s average)	4050	1958 (48.3)	0.000
	Low (lower than the province’s average)	4060	946 (23.3)	
	Social economic situation among the sampled cities			
	Grade I (GDP per capita higher than 60,000 yuan)	1311	820 (62.5)	0.000
	Grade II (GDP per capita from 30,001 to 40,000 yuan)	1961	1034 (52.7)	
	Grade III (GDP per capita from 20,001 to 30,000 yuan)	2744	809 (29.5)	
	Grade IV (GDP per capita lower than 10,000 yuan))	2094	241 (11.5)	
	Population density of the target cities			
	High (more than 801 per km^2^)	2671	1240 (46.4)	0.000
	Middle (from 401 to 800 per km^2^)	2624	1137 (43.3)	
	Low (less than 400 per km^2^)	2815	527 (18.7)	
	Number of health technical personnel per 1000 population of the provinces			
	High (more than 4)	2725	1516 (55.6)	0.000
	Middle (from 3.2 to 3.4)	2548	351 (13.8)	
	Low (less than 3)	2837	1037 (36.6)	
Predisposing factors (Individual level)				
	Age	26.97 ± 4.64		-
	Position of residence			
	Urban	3011	1729 (57.4)	0.000
	Rural	5099	1175 (23.0)	
	Ethnicity			
	Han	7410	2797 (37.7)	0.000
	Minority	700	107 (15.3)	
	Educational level of the respondents			
	Less than primary school (6 years or less)	784	96 (12.2)	0.000
	Middle school (9 years)	3724	806 (21.6)	
	High school or equivalent (12 years)	1745	755 (43.3)	
	College (14 years)	1107	678 (61.2)	
	More than Bachelor's (16 years or more)	750	569 (75.9)	
	Educational level of the respondents’ spouse			
	Less than primary school	392	50 (12.8)	0.000
	Middle school	3531	699 (19.8)	
	High school or equivalent	2061	774 (37.6)	
	College	1056	607 (57.7)	
	More than Bachelor's	1070	774 (72.3)	
	Occupation of the respondents			
	The unemployed	1441	549 (38.1)	0.000
	Employee	5086	2192 (43.1)	
	Peasant	1583	163 (10.3)	
	Occupation of the respondents' spouse			
	The unemployed	318	124 (39.0)	0.000
	Employee	6667	2675 (40.1)	
	Peasant	1125	105 (9.3)	
	Migrant			
	Migrant population	1870	726 (38.8)	0.002
	Not migrant population	6240	2178 (34.9)	
	Attending maternal preparation classes			
	Yes	3163	1634 (51.7)	0.000
	No	4947	1270 (25.7)	
Enabling factors (Individual level)				
	Insurance			
	Insured	5981	2067 (34.6)	0.000
	Uninsured	2129	837 (39.3)	
	Income per person, per month in a family (yuan, RMB)			
	0–1200	3752	719 (19.2)	0.000
	1200.01–2400	2688	1043 (38.8)	
	2400.01	1670	1142 (68.4)	
	Accessibility of health information			
	Very easy	1388	707 (50.9)	0.000
	Not too difficult	4798	1840 (38.3)	
	Somewhat difficult	1631	328 (20.1)	
	Quite difficult	205	24 (11.7)	
	Very much difficult	88	5 (5.7)	
Enabling factors (health care institution level)				
	Levels of Health care institutions for delivery			
	Primary health centers	1410	336 (23.8)	0.000
	Secondary health centers	4037	1236 (30.6)	
	Tertiary health centers	2264	1260 (55.7)	
	Other health centers	399	72 (18.0)	
	Types of Health care institutions for delivery			
	General hospitals	3538	894 (25.3)	0.000
	MCH centers	4091	1856 (45.4)	
	Community health center/ township health centers	244	47 (19.3)	
	Other health centers	237	107 (45.1)	
Need factors (Individual level)				
	Age risk			
	<20 years	148	24 (16.2)	0.000
	20.01–34.99 years old	7433	2760 (37.1)	
	≥35 years old	529	120 (22.7)	
	Self-evaluation of health during pregnancy			
	Very good	2647	1049 (39.6)	0.000
	Good	3658	1351 (36.9)	
	General	1695	474 (28.0)	
	Bad	103	28 (27.2)	
	Very bad	7	2 (28.6)	
Health behavior (Individual level)				
	The frequency of routine prenatal checkups			
	Less than 4 times	2293	331 (14.4)	0.000
	5 to 7 times	2538	577 (22.7)	
	More than 8 times	3279	1996 (60.9)	
	Knowledge about DS			
	Enough	2049	1265 (61.7)	0.000
	Limited	6061	1639 (27.0)	

A comparison of questionnaire data with population data indicated no statistical differences in ethnicity, education-level distribution, and the average income of an urban population. However, the average income of the rural population in the survey was higher than that recorded for the population as a whole (Table 2).

**Table 2:** The representativeness of the sample

	The study sample	Whole population	Statistical calculators	*P*
Ethnicity distribution				
Minority (%)	8.63	8.40	0.56^a^	0.45
Education level				
Middle school and lower (%)	55.58	50.12	0.24^a^	0.62
Personal annual income				
Urban area(yuan)	15256.8	13786	0.43^b^	0.67
Rural area(yuan)	5227.8	4140	33.41^b^	0.00

^a^Chi-square test

^b^ *T*-test

### MSS utilization and its associated factors

Of the 8110 women surveyed, 2904 (35.8 %) used MSS prenatally of their current delivery. The uptake rate varied from 6.5 % to 68.2 % for the different cities, and the median uptake rate among the sampled cities was 18.4 %.

The results of the univariate analysis were used to identify candidate explanatory variables for use in the multilevel models. The variables associated with MSS utilization from the univariate analysis included contextual factors, predisposing factors, enabling factors, need factors and factors related to respondents’ health behavior (Table 1).

Multilevel logistic regression analysis with the dependent variable of whether to use MSS was performed to reveal the factors associated with MSS uptake (Table 3, model 1). The results showed that the respondents' socioeconomic situation, demographic characteristics (including age, urban–rural residence, occupation and education experience, and residential status), health belief, frequency of routine prenatal checkups and health knowledge all played an active part in their decision whether to undergo MSS. The women who did not attend maternal preparation classes (OR 0.70, 95 % CI 0.61–0.81), those who received less than seven prenatal checkup appointments (OR 0.40, 95 % CI 0.34–0.46), and those who had limited DS knowledge (OR 0.46, 95 % CI 0.39–0.54) were less likely to use MSS than their counterparts. Support from health facilities was also associated with MSS uptake. Women giving birth in primary and secondary institutions, where they had usually obtained prenatal care, were less likely to use MSS compared with those giving birth in tertiary healthcare institutions (OR 0.38, 95 % CI 0.19–0.76 versus OR 0.57, 95 % CI 0.33–0.96). Furthermore, women 35 years or older were less likely to use MSS compared with those aged 20 to 35 years (OR 0.47, 95 % CI 0.35–0.62).

**Table 3:** Multilevel logistic regression analysis of associated factors of the outcome and intermediate factors (*n* = 8110)

	Model1		Model 2		Model 3		Model 4	
Dependent Variables	MSS utilization or not		Knowledge about DS		Frequency of routine prenatal checkups		Attending maternal preparation classes or not	
Definition	1 = yes		1 = enough		1 = more than eight		1 = yes	
	0 = no (ref.)		0 = limited (ref.)		0 = less than seven (ref.)		0 = no(ref.)	
Accuracy	83.1 %		80.3 %		79.6 %		74.6 %	
Model	OR [95 % CI]	*P*-value	OR [95 % CI]	*P*-value	OR [95 % CI]	*P*-value	OR [95 % CI]	*P*-value
Social economic situation of the sampled cities (categorical)								
1 = Grade I (high)	13.73[2.64–71.31]	0.002	1.68[0.48–5.89]	0.418	6.18[0.56–68.15]	0.137	2.76[0.84–9.06]	0.094
2 = Grade II	5.06[1.12–22.81]	0.035	1.38[0.43–4.39]	0.588	5.13[0.59–44.63]	0.139	2.96[0.99–8.89]	0.053
3 = Grade III	2.11[0.53–8.52]	0.292	1.00[0.35–2.89]	0.998	7.03[0.938–52.67]	0.058	1.73[0.63–4.73]	0.287
4 = Grage IV (low, ref.)								
Age groups of the respondents (categorical)								
1 = younger than 20 years old	0.67[0.39–1.15]	0.142	0.47[0.25–0.87]	0.017	0.43[0.26–0.71]	0.001	0.51[0.32–0.81]	0.005
2 = 35 years or older	0.47[0.35–0.62]	0.000	0.78[0.60–1.01]	0.059	0.78[0.61–1.00]	0.048	0.94 [0.76–1.17]	0.593
3 = from 20 to 35 years old (ref.)								
Respondent’s residence (categorical)								
0 = rural area	0.69[0.58–0.81]	0.000	0.88[0.75–1.04]	0.121	0.70[0.56–0.81]	0.000	0.63[0.54–0.72]	0.000
1 = urban area (ref.)								
Ethnicity of the respondents (categorical)								
1 = Han	1.23[0.91–1.67]	0.182	0.94[0.71–1.23]	0.647	0.87[0.66–1.14]	0.303	0.96[0.74–1.23]	0.730
2 = Minority (ref.)								
Educational level of the respondents (categorical)								
1 = less than primary school	0.41[0.27–0.60]	0.000	0.19[0.13–0.28]	0.000	0.26[0.19–0.38]	0.000	0.24[0.18–0.33]	0.000
2 = middle school	0.49[0.37–0.65]	0.000	0.36[0.28–0.46]	0.000	0.51[0.39–0.66]	0.000	0.38[0.31–0.48]	0.000
3 = high school or equivalent	0.62[0.47–0.81]	0.000	0.59[0.47–0.74]	0.000	0.74[0.58–0.95]	0.018	0.59[0.48–0.73]	0.000
4 = college	0.74[0.56–0.97]	0.027	0.77[0.62–0.96]	0.018	0.94[0.73–1.21]	0.623	0.72[0.58–0.89]	0.003
5 = more than bachelors’ (ref.)								
Occupation of the respondents (categorical)								
1 = unemployed	1.63[1.27–2.09]	0.000	1.14[0.92-1.42]	0.243	0.86[0.70–1.06]	0.157	1.46[1.22–1.75]	0.000
2 = employee	1.44[1.10–1.89]	0.009	1.11[0.86-1.43]	0.420	0.90[0.71–1.14]	0.381	1.03[0.83–1.28]	0.773
3 = peasant (ref.)								
Migrant situation of the respondents (categorical)								
0 = migrant population	0.59[0.50–0.69]	0.000	1.05[0.90–1.22]	0.572	0.83[0.71–0.96]	0.015	0.58[0.50–0.66]	0.000
1 = not migrant population (ref.)								
Health belief - attending maternal preparation class or not (categorical)								
0 = not attending maternal preparation class	0.70[0.61–0.81]	0.000	0.62[0.54–0.71]	0.000	0.66[0.57–0.75]	0.000		
1 = attending maternal preparation class (ref.)								
Health insurance coverage situation of the respondents (categorical)								
0 = uninsured	0.92[0.79–1.07]	0.271	0.84[0.73–0.97]	0.020	0.88[0.76–1.02]	0.081		
1 = insured (ref.)								
Income per person, per month in a family (yuan) (categorical)								
1 = from 0 to 1200	0.86[0.70–1.05]	0.147	1.00[0.82–1.21]	0.969	0.52[0.43–0.63]	0.000		
2 = from 1200.01–2400	0.95[0.80–1.14]	0.609	1.05[0.89–1.25]	0.560	0.70[0.59–0.83]	0.000		
3 = 2400.01 and above (ref.)								
Self-evaluation of health information accessibility (categorical)								
1 = very easy	0.80[0.24–2.63]	0.710	3.28[0.65–16.59]	0.152	3.71[0.80–17.15]	0.093		
2 = not too difficult	0.70[0.22–2.30]	0.561	3.16[0.63–15.88]	0.164	3.07[0.67–14.11]	0.149		
3 = somewhat difficult	0.60[0.18–1.97]	0.402	1.77[0.35–8.93]	0.492	2.02[0.44–9.31]	0.366		
4 = quite difficult	0.52[0.14–1.87]	0.315	2.09[0.38–11.41]	0.393	1.13[0.23–5.65]	0.885		
5 = very much difficult								
Levels of health care institutions where the respondents found (categorical)								
0 = other health centers	0.47[0.12–1.88]	0.285	1.45[0.40–5.26]	0.576	0.93[0.29–3.00]	0.903		
1 = primary health centers	0.38[0.19–0.76]	0.006	1.01[0.52–1.96]	0.976	0.65[0.36–1.17]	0.152		
2 = secondary health centers	0.57[0.33–0.96]	0.035	1.08[0.65–1.79]	0.766	0.73[0.47–1.13]	0.158		
3 = tertiary health centers(ref.)								
Types of health care institutions where the respondents found(categorical)								
0 = other health centers	0.46[0.08–2.56]	0.377	1.35[0.25–7.17]	0.727	3.74[0.77–18.08]	0.101		
1 = general hospitals	0.22[0.05–1.02]	0.052	0.10[0.23–4.42]	0.997	3.24[0.78–13.54]	0.107		
2 = maternal and child health centers	0.38 [0.08–1.88]	0.235	1.59[0.34–7.38]	0.557	4.29[0.99–18.60]	0.052		
3 = community health center or township health centers (ref.)								
Self-evaluation of health during pregnancy of the respondents (categorical)								
1 = very good	0.39[0.04–3.43]	0.392	0.30[0.04–2.50]	0.268	2.43[0.20–30.10]	0.490		
2 = good	0.41[0.46–3.67]	0.426	0.26[0.03–2.17]	0.215	2.05[0.17–25.35]	0.577		
3 = general	0.40[0.04–3.53]	0.407	0.25[0.03–2.03]	0.193	2.11[0.17–26.21]	0.561		
4 = bad	0.29[0.03–2.77]	0.283	0.33[0.04–2.88]	0.313	2.20[0.17–29.90]	0.548		
5 = very bad (ref.)								
Frequency of routine prenatal checkups (categorical)								
0 = less than 7	0.40[0.34–0.46]	0.000	0.66[0.57–0.77]	0.000				
1 = more than 8 (ref.)								
Knowledge of DS (categorical)								
0 = limited	0.46[0.39–0.54]	0.000						
1 = enough (ref.)								

According to Andersen’s behavioral model, the main effect flow is from contextual characteristics to individual ones, which include predisposing, enabling, and need factors, and to health behavior factors, with corresponding feedback loops. Among health behavior factors, personal health practice provides the basic effect, which then affects health service utilization through the healthcare process. Based on the results of model 1, further focus was placed on the respondents’ health knowledge, uptake of routine prenatal checkups, and health beliefs to explore approaches to improve MSS utilization. Three additional multilevel logistic regression models were performed to discover the associated factors of these three intermediate factors. These three factors were designated as dependent variables, and the prevariables found meaningful in model 1 according to Andersen’s behavioral model were chosen as their independent variables.

The results of the additional three analyses indicated that the respondents’ health beliefs and routine prenatal checkup utilization were factors associated with participants’ health knowledge, and the respondents’ health belief was a factor associated with their use of routine prenatal checkups. Among other predisposing factors in all three models were the effects of education and age group, with less-educated women having lower health beliefs, less health knowledge, and attending fewer routine prenatal checkups. Among enabling factors, the effect of the level of healthcare institution that the participants attended was not found to be a factor in their routine prenatal checkup utilization or health knowledge (Table 3).

## Discussion

The purpose of this study was to identify factors affecting the uptake of MSS by Chinese pregnant women so that suitable policies can be developed to ensure MSS is equally accessible to the whole population of mainland China.

From the univariate analysis, all of the factors chosen according to Andersen’s behavioral model were associated with MSS utilization, although some were confounding or indirect factors. Four multilevel regression models were applied stepwise to the data, according to the flow of factors of Andersen’s behavioral model. Major associated factors found through the univariate analysis were included. The results of the multilevel regressions were consistent with Andersen’s behavioral model. All five kinds of factors (contextual, predisposing, enabling, need, and health behavior) from Andersen’s behavioral model were found to be associated with MSS utilization. Aside from the respondents’ demographic characteristics, three intermediate factors, the respondents’ number of prenatal checkups, DS knowledge, and attendance at maternal preparation classes all played an active role in their MSS uptake decision. MSS utilization was also affected by the respondents’ prenatal care service providers. When the three intermediate factors were chosen as outcome variables to fit a further three regression models, the respondents’ education level was a key demographic factor associated with all four outcome variables.

Combining the results of the four models revealed that insufficient education is the single most important demographic factor for MSS underutilization. Also, regular prenatal checkups, health knowledge and attendance at maternal preparation classes were associated with greater MSS uptake, as were the establishment of a positive relationship between the participant and her prenatal care providers, while this relationship was independent of the relationship found above.

Uptake rates for MSS in mainland China are lower compared with the uptake of prenatal screening in other countries (35 % in 1993 and more than 50 % since 2007 for the United Kingdom [22, 23], 44 % in 2004 for Australia [24], 50 % in 1992 and 72 % in 2011–2012 for the United States [25], 84 % in 2006 for Denmark [26]). The experience of the above countries showed that progress in increasing the prenatal screening rate stemmed from using advanced technology and employing effective health policies [27–29]. China introduced MSS technology about 10 years after developed countries. Besides the regulations on technology administration and the national standard operating protocol for MSS and invasive diagnostic test delivery (mainly as clinical practice guidelines) published in 2003 and 2010, there have been no other specific national policies to support better access to MSS and invasive diagnostic tests.

The uptake rate of MSS (23 % of the combined tests between 2009 and 2011 [13]) in The Netherlands was lower than that found in mainland China. The Netherlands maintain a more uniform healthcare system and have better prenatal screening accessibility than China. The screening technology was introduced to the two countries at the same time. China may have a higher uptake rate for MSS than The Netherlands because the combined test was not routinely provided in The Netherlands but was in some healthcare institutions in China. Similar to the experience of other developed countries [14, 16, 30, 31] and earlier research of the Zhejiang data [32], there are regional variations in MSS utilization in China.

Earlier studies from other countries have shown that the factors associated with uptake of MSS and further diagnostic procedures include individual and external factors. Individual factors include age, parity, socioeconomic status, ethnicity, and religion; external factors include local health system and policy arrangement, and providers’ characteristics and attitude [33–39]. In China, more attention is given to the vulnerable population when focusing on essential public health service utilization. Factors including age, gender, education, occupation, insurance status, income, capacity of service providers, and health resource distribution were taken into consideration when suggestions for equitable access were discussed [6]. Similar results regarding the key factors to consider were obtained in this study.

Issues relevant to prenatal test uptake include income and the effect of insurance coverage. In this study, the respondents’ insurance status was not found to be a factor associated with MSS uptake rate, although a review of the Chinese literature found social health insurance was considered a significant enabling factor of service utilization. Prior to the launch of new health system reforms in 2009, MSS was not covered by social health insurance, and most users had to pay for it themselves. The prices then of MSS and further invasive tests were 120 Yuan RMB and over 1000 Yuan RMB, respectively. However, at that time, there was no association between family income and MSS utilization, which indicated that the cost of the test was not the key factor in test underutilization. This suggests that some pregnant women missed MSS not because of financial reasons, but because they were not offered the test—or if the service was provided, the pregnant women may have simply opted out of screening, no matter the price or insurance coverage.

The inverse relationship between age and MSS utilization found in this study was unexpected. In this study, respondents’ age can be treated as either a predisposing factor or a need factor. Older women have a higher risk of having a child affected by a chromosomal disorder, such as DS, than younger childbearing women, so those older than 35 years should be more likely to seek MSS and further diagnostic tests. However, results of this study showed the opposite. One possible reason was that some respondents were recommended to have invasive diagnostic tests directly. Similar studies published in other countries have shown that it was difficult to draw firm conclusions as to the principal factors influencing older women’s uptake of prenatal screening and diagnostic services, and more research is needed [40]. There may be instances such as an older pregnant woman who had already had one healthy baby feeling immune to the increased risk of BD, so not seeking MSS. Miscarriage risk from invasive diagnostic tests could be another potential explanation for lower test uptake rate. Presently, there is not enough related information to draw a definitive conclusion.

Last, the effect of service providers should be noted, as shown by Gitsels-van der Wal et al. who reported a different uptake rate of the combined test among different midwifery practices [13]. Among three models when providers’ levels were included, its effect can only be found in model 1 where MSS utilization was the outcome variable, and women delivered in high-level healthcare institutions were more likely to use MSS. Results of the other two models (models 2 and 3) indicated that there was no relationship between respondents’ health knowledge, frequencies of routine prenatal checkups and service facilities’ level. The process and content of routine prenatal checkups is regulated in China, and all health facilities providing prenatal checkups must meet basic requirements. MSS is treated as a highly technical procedure and only a proportion of health facilities are able to reach the required standard set by the MOH to provide it. Most of the prenatal diagnostic centers who did reach the required standard were tertiary or secondary healthcare institutions. Women who received routine prenatal checkups from other health facilities would probably miss MSS unless there was a network of services that could be accessed.

The limitations of this research should also be mentioned. It is unlikely that the widely differing utilization rates across the country can be explained by variation in individual values alone. Although data on the level, type, and location of healthcare institutions where the respondents delivered (and also received their prenatal care service generally) were collected, detailed information about healthcare resources, and in particular whether the facility could deliver MSS or invasive diagnostic tests, was not collected. Gaps in service capacity and capability among different healthcare institutions could be very important for service utilization. The results of our study show that tertiary healthcare institutions play an active role in MSS and further diagnostic test utilization.

From this research, two policy strategies can be implemented to ensure that all women have equal access to prenatal screening. First, preferential policy strategies should be developed for the vulnerable population. Less-educated women should be provided with enough resource support to help them build their health beliefs, improve their personal health practice, acquire health knowledge and use MSS.

Second, policy strategies on MSS delivery should be developed. International experience indicates that a primary healthcare-centered integrated delivery model was effective and efficient in MSS and further diagnostic test delivery [41]. Till recently, MSS delivery in China is mainly through tertiary healthcare institutions or prenatal diagnostic centers. Service networks are limited, which warrants a change in policy. The transformation of MSS delivery should improve regional variation on MSS uptake rate, as long as the primary healthcare is of sufficient quality, and its responsibility is identified [42].

Chinese health system reforms in 2009 announced a strong Government responsibility for population health and a state-funded public health system. Better health support and policy arrangements, for example social health insurance coverage for the prevention of birth defects and free screening for high-risk pregnant women, will be highlighted.

At the start of 2015, the first 109 healthcare institutions were granted permission to deliver noninvasive prenatal tests (NIPT) from the MOH of China. Although there was not a large sample report in China about its sensitivity and specificity, the risk of miscarriage with NIPT was definitely lower than with MSS plus an invasive diagnostic procedure. The barriers to accessing prenatal screening services found in the present study should also be applied to NIPT uptake.

Further research might expand on this study by combining the providers’ characteristics with the users’ to gather more information.

## Conclusion

The associated factors identified in this study, including contextual, predisposing, enabling, need, and health behavior factors, were largely consistent with Andersen’s behavioral model of health services utilization (with some exceptions unique to Chinese culture). Health policies should mainly focus on two aspects to improve MSS utilization. First, less-educated pregnant women should be treated as the most urgent target population and be provided with enough support to improve their health beliefs, personal health practice and health knowledge. Second, MSS delivery should be reorganized to make it primary healthcare-centered and more integrated.

## Abbreviations

BD: Birth defects
DS: Down syndrome
GDP: Gross domestic product
LMIC: Low- and middle-income countries
MCH: Maternal and child health
MOH: Ministry of Health
MSS: Maternal serum screening
NIPT: Noninvasive prenatal test
PRC: People’s Republic of China
WHO: World Health Organization
